# Social Context Disambiguates the Interpretation of Laughter

**DOI:** 10.3389/fpsyg.2017.02342

**Published:** 2018-01-12

**Authors:** William Curran, Gary J. McKeown, Magdalena Rychlowska, Elisabeth André, Johannes Wagner, Florian Lingenfelser

**Affiliations:** ^1^School of Psychology, Queen’s University Belfast, Belfast, United Kingdom; ^2^Human-Centered Multimedia, Institut für Informatik Universität Augsburg, Augsburg, Germany

**Keywords:** laughter, social interaction, social context, laughter interpretation, non-verbal communication

## Abstract

Despite being a pan-cultural phenomenon, laughter is arguably the least understood behaviour deployed in social interaction. As well as being a response to humour, it has other important functions including promoting social affiliation, developing cooperation and regulating competitive behaviours. This multi-functional feature of laughter marks it as an adaptive behaviour central to facilitating social cohesion. However, it is not clear how laughter achieves this social cohesion. We consider two approaches to understanding how laughter facilitates social cohesion – the ‘representational’ approach and the ‘affect-induction’ approach. The representational approach suggests that laughter conveys information about the expresser’s emotional state, and the listener decodes this information to gain knowledge about the laugher’s felt state. The affect-induction approach views laughter as a tool to influence the affective state of listeners. We describe a modified version of the affect-induction approach, in which laughter is combined with additional factors – including social context, verbal information, other social signals and knowledge of the listener’s emotional state – to influence an interaction partner. This view asserts that laughter by itself is ambiguous: the same laughter may induce positive or negative affect in a listener, with the outcome determined by the combination of these additional factors. Here we describe two experiments exploring which of these approaches accurately describes laughter. Participants judged the genuineness of audio–video recordings of social interactions containing laughter. Unknown to the participants the recordings contained either the original laughter or replacement laughter from a different part of the interaction. When replacement laughter was matched for intensity, genuineness judgements were similar to judgements of the original unmodified recordings. When replacement laughter was not matched for intensity, genuineness judgements were generally significantly lower. These results support the affect-induction view of laughter by suggesting that laughter is inherently underdetermined and ambiguous, and that its interpretation is determined by the context in which it occurs.

## Introduction

Because of its ubiquitous nature laughter has become a recent focus of research across a range of scientific disciplines. The thesis that it is an evolutionarily ancient behaviour preceding spoken language is supported by reports of laughter-like behaviour in non-human primates (Davila Ross et al., 2009, Davila-Ross et al., 2011) and of similarities in acoustic elements of laughter in humans and play vocalisations of other primates (Vettin and Todt, 2005). As in human infants (Rothbart, 1973) laughter-like behaviour in chimpanzees prolongs play actions (Matsusaka, 2004), suggesting that laughter is an important tool for promoting social affiliation and developing cooperative and competitive behaviours (Davila-Ross et al., 2011). Thus from an evolutionary perspective laughter can be viewed as a key adaptive behaviour because of its facilitative effect on social cohesion.

Just as speech follows a set of rules so too, it seems, does laughter, with conversation analysis uncovering a number of rules of laughter behaviour (Glenn, 2003; Holt, 2010, 2011). For example in dyadic conversations the speaker is more likely than the listener to laugh first, while in group conversations the listeners are more likely to laugh first (Glenn, 2003). Laughter also has a regulatory function in conversations by serving as a turn-taking cue or signalling that the speaker may be approaching a transition point in his/her utterance (O’Donnell Trujillo and Adams, 1983; Holt, 2010; Bonin et al., 2012).

Neuropsychological research has revealed the existence of two partially dissociable neural pathways underlying two different types of laughter – spontaneous and volitional. One pathway is emotionally driven and involuntary, arising in subcortical, limbic, and brainstem areas and culminating in a “laughter-coordinating” centre in the dorsal upper pons; the other is a voluntary motor pattern that originates in frontal premotor areas and directly influences the motor cortex. Damage to the former pathway inhibits the production of spontaneous (but not volitional) laughter production, and damage to the latter pathway inhibits volitional (but not spontaneous) laughter production (Wild et al., 2003). Spontaneous laughter and volitional laughter do not only arise from separate neural systems but also appear to be processed in distinct brain regions, as evidenced by increased amPFC and anterior cingulate cortex activity when participants listen to volitional laughter as opposed to spontaneous laughter (McGettigan et al., 2013). McGettigan et al. (2013) suggest that volitional laughter induces a stronger engagement of mentalising processes, and postulate that this is indicative of attempts to assess the emotional state and intentions of the laugher.

The conclusions drawn by McGettigan et al. (2013) speak to a long-standing debate (Fridlund, 1994; Parkinson, 2005; Feldman Barrett, 2006, 2017) on the function of social signals and emotions – do they merely indicate the expresser’s felt state or can they function as active socio-communicative instruments? Laughter, as one of our most common non-verbal social signals, is well placed to illuminate this debate. According to the mainstream view, laughter conveys information about the laugher’s underlying emotional state and listeners can decode this information to gain knowledge about other people’s feelings and motivations. This ‘representational’ approach, where signals are referential and “about” something, often relies on the “conduit metaphor” (Reddy, 1979; Rendall et al., 2009), according to which signals transmit information from and about their sender and convey it to a receiver who then decodes the message. The ‘representational’ view of laughter fits with our everyday use of various types of laughter, such as joyful laughter, schadenfreude laughter, taunting laughter, and embarrassed laughter: the laughter indicates the expresser’s internal state and serves as a “readout” of the laugher’s current emotions. If the function of social signals is to represent an internal felt state then discrete representational coding provides an efficient indication of felt state. These internal states should be relatively easy to distinguish and there would be distinct laughter signals that distinguish each discrete “natural kind” of felt state (Feldman Barrett, 2006). Coming back to the examples above, distinct kinds of laughter should accompany feelings of joy, embarrassment, or schadenfreude. However, the lack of evidence for affect-specific laughter types challenges the notion that laughter is used to communicate underlying emotional states. It may be that in these “joint state” cases natural kinds are not represented, that is there is no discrete or specific “nervous laughter” or “schadenfreude laughter,” but there is an additive combination of social signals. For example, some signals may be to do with nervousness and the laughter signal may have a different communicative function. An alternative theoretical view is that, rather than passively transmitting information about the laugher’s emotional state, laughter is used to influence the affective states of listeners (Owren and Bachorowski, 2003). To differentiate between the representational and affect-induction views of laughter, Owren and Bachorowski use the analogy of a crying baby. According to the representational account, the crying merely delivers appropriate encoded information about the unpleasant feelings that the baby is experiencing and should stop when the information is provided. An affect-induction view, on the other hand, holds that the acoustic and visual qualities of crying induce negative affect in a listener that persists until the problem is resolved. Similarly, in the case of primate vocalisations, “the primary function of calling is to influence listener attention, arousal, and emotion rather than to transmit information.” Adult human communication, however, is more complex than a baby’s cry or primate calls and is often accompanied by verbal messages.

According to Owren and Bachorowski (2003), laughter is also a form of affect-induction communication. As evidence against the representational view of this behaviour, they point to its generalizability and lack of specificity. Namely, the same laugh may be used to induce positive or negative affect in a listener, and the interpretation of the same “laugh episode” will be determined by a number of factors, including the behaviours the laughter accompanies, the relationship between the laugher and listener, or the listener’s emotional state when hearing the laughter. Thus, while in one context a listener may experience a laugh as derisory, the same laugh may become a positive experience in a different context–as Papousek et al. (2014) have shown.

The key contrasts between these two approaches are: (1) the nature of the motivation for the behaviour – in the representational case the laugher passively indicates their felt state, in the affect-induction case laughter is used to influence the receiver’s affect; (2) the level of ambiguity – in the representational case different laughter types would facilitate distinguishing between felt states, in the affect-induction case laughter would be used in conjunction with other social signals and contextual factors to induce a desired affect. Furthermore, the laughter would be ambiguous in the absence of social context.

Here, we propose a modified version of the affect-induction approach. From the affect-induction perspective affect is induced through the combination of laughter with additional factors that dynamically unfold throughout the course of a social interaction – such as verbal information, social context, or knowledge of the listener’s emotional state. These factors influence the receiver and their accurate understanding ensures that the laughter is interpreted in accordance with the expresser’s socio-communicative goals. In this view, a laugh by itself is an underdetermined and ambiguous social signal. In other words, hearing or viewing somebody laughing without additional information does not provide enough information to be sure of their emotional state. Our modified version of the affect-induction approach takes into account the role of intensity in laughter communication.

Intensity is an important communicative component of many social signals and emotional expressions (Banse and Scherer, 1996; Bänziger and Scherer, 2005; Biele and Grabowska, 2006), and also appears to play an important role in laughter (Darwin, 1872; van Hooff, 1972; Preuschoft and van Hooff, 1997, McKeown et al., in preparation). It adds a level of complexity to our distinctions between representational views and affect-induction views. Given that voiced laughter produces stronger affect-related responses in a listener relative to unvoiced laughter (Bachorowski and Owren, 2001), it follows from the perspective of the affect-induction approach that different laughter intensities should vary in the extent to which they induce affect in the listener; the representational model, on the other hand, would predict that differing laughter intensities would indicate different levels of a given felt emotion. It is probable that laughter intensity reflects a complex interplay between felt emotion and contextual influences on affect induction, in the same way that emotional facial expressions are influenced by both these factors (Fridlund, 1994). However, apart from components of the laughter signal that make a laugh more or less intense, we argue (as others have argued; e.g., Russell et al., 2003) that there are no morphological or acoustic markers of laughter that contain meaning in a representational sense. In other words, there is not a one-to-one relationship between a felt emotion and laughter produced while experiencing that emotion. Take the following scenario as an example. Two people, James and Robin, are having an intense argument while being observed by a neutral group. James makes a witty comment that highlights a central flaw in Robin’s argument. The people observing the argument laugh in response to the witty comment. Both participants in the argument will hear the same laughter, but are likely to interpret it differently; James will interpret the laughter as humorous and an appreciation of his wit while Robin is likely to interpret it as derisory laughter. In this scenario identical laughter is taken to signal two very different emotions – humour and derision. This would not occur if different emotions were represented by laughter with distinct acoustic markers. Rather, we propose that laughter is intrinsically underdetermined and ambiguous, and a listener’s interpretation of laughter is determined by factors such as the context in which it occurs. The same view has been taken by Owren and Bachorowski’s affect-induction approach. However, the modified affect-induction model differs from the original model in one key respect. While both models would agree that its inherent ambiguity allows laughter from different interactions to be wholly interchangeable without affecting apparent genuineness of an interaction, the modified version proposes that this interchangeability is restricted to laughter of similar intensity.

Using a novel experimental approach to test our hypothesis, we reasoned that participants’ judgements of social interactions should be unaffected if the laugh response in an original dyadic interaction is replaced with a similar intensity laugh response from a different part of the same interaction. In contrast, if a high intensity laugh response is replaced with a low intensity laugh response, or vice versa, there should be a measurable reduction in ‘genuineness’ ratings. Interchangeability of laughter would be taken as evidence supportive of the affect-induction model’s central tenet that laughter is an inherently ambiguous signal; and evidence of intensity-specific interchangeability would support the modified affection-induction model’s incorporation of intensity as an important factor in interpreting laughter. If, however, exchanging laughter always results in an interaction seeming less genuine, this will be taken as support for the representational model.

## Materials and Methods

### Overview

Here we report on two experiments, in which participants viewed video sequences displaying two persons. Each sequence involved a ‘listener’ laughing in response to something a ‘story-teller’ said. Participants then judged how real or genuine the interaction was, that is how confident they were that the interaction actually took place. The recorded interactions contained either high intensity or low intensity laughter. To differentiate the story-teller from the laugher we refer to the story-teller as producing a high or low intensity “laughable context,” and the laugher produces either a high or low intensity “laugh response.”

### Stimuli

Stimuli were generated from interactions created as part of the ILHAIRE laughter database (McKeown et al., 2015) using a naturalistic story telling task. The Social Signal Interpretation framework (Wagner et al., 2013) was used to capture video and audio information of groups of two or more people. The task was designed to exert minimal influence on the behaviours of the interlocutors as they conversed with one another, while allowing the synchronised capture of high quality audio and video material.

### Intensity Selection

Laugh stimuli were extracted from the original interactions and rated by participants along a number of dimensions including laugh intensity and humour, with participants recruited using Amazon’s Mechanical Turk. An informed consent form explaining the study’s procedure and the experimenter’s contact details was placed at the very beginning of the ratings form. MTurk participants were informed that their identity would remain confidential and that they could withdraw from the experiment at any time by simply logging out before completing the rating exercise. An incomplete data set from a participant was interpreted as the participant deciding to withdraw from the experiment, and the relevant data were destroyed. Analysis of 9421 ratings of 870 laugh episodes revealed a strong relationship between the intensity of a laugh and how much people judged it to be related to humour (see McKeown and Curran, 2015, for more detail).

The stimuli used in the following experiment are taken from two conversational partners participating in an interaction between three people that lasted for 70 min. Since both social context and intensity affect emotion perception (Fridlund, 1994), for this initial experiment we only used the recordings of two conversational partners who were both male, who shared the same cultural background, and who were friends. According to Owren and Bachorowski (2003) male friend pairs should produce high rates of laughter that are acoustically extreme in both pitch and duration. The naturalistic form of data gathering has the effect of producing natural laughter with greater ecological validity; however, it also means that many of the laugh instances must be excluded from the experiment as they include other verbal cues (e.g., speech) and non-verbal cues (e.g., not looking at the speaker, covering the face with a hand) not related to the effect of interest.

The laughter selected for intensity rating was laughter in which there was an unobstructed frontal view of the laugher’s face, the laugher was looking at the speaker, and there was no speech during laughter. Laughter was rated for intensity using the question “Can you rate the intensity of the laugh on a 10 point scale, from 1 no intensity to 10 maximum intensity?” This rating strategy assumes the laugh rater has a degree of expertise in laughter through being a lifetime observer of laughter and consequently minimal instructions are provided to avoid leading the rater into a particular interpretation of the concept of laugh intensity. Laughter stimuli assigned to the lowest quartile of intensity ratings were designated as ‘low intensity’ laughter, and laughs assigned to the highest quartile were designated as ‘high intensity’ laughter. The corresponding laughable contexts were not independently rated, but are termed high and low intensity laughable contexts by virtue of the fact that they resulted in high or low laugh responses. After the exclusion of laughter instances that contained verbal and non-verbal cues not related to the effect of interest, 8 laugh responses combined with their “laughable” context remained – 4 high intensity and 4 low intensity. These were used to generate the experimental stimuli.

### Stimulus Generation

**FIGURE 1:**
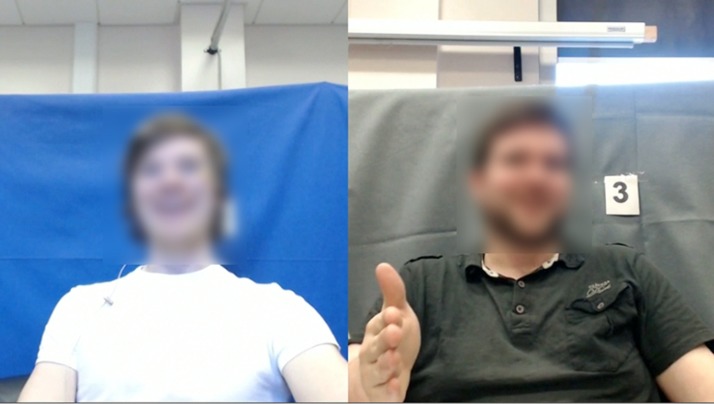
Screenshot of the interaction stimuli. The story teller and listener were positioned on the right and left, respectively. Each interaction lasted approximately ten seconds, during which time the unfolding story led up to the laughter event. The listener’s audio–visual stream was frozen at a frame containing a neutral facial expression up until the point where the laugh response began. Faces were not blurred during the actual experiment.

Each laugh and laughable context was placed alongside each other on a computer screen to produce a reconstruction of the interaction (see **Figure 1**). We generated stimuli for six conditions: two control conditions containing both the high and low intensity original interactions; two same intensity conditions, one containing high intensity laughable contexts with swapped high intensity laugh responses and another containing low intensity laughable contexts with swapped low intensity laugh responses; and two opposite intensity conditions, one containing high intensity laughable contexts with swapped low intensity laugh responses and another containing low intensity laughable contexts with swapped high intensity laugh responses. The listener’s video stream was frozen at a frame containing a neutral facial expression up until the point where the laugh response began; this was to avoid unwanted social cues interfering with the participants’ perception of the laugh.

In each experiment we adopted a 2 (laugh response intensity) × 3 (laughable context) design. Laugh responses had two levels, high or low intensity. Laughable context had three levels, one control condition (original video) and two experimental conditions (same intensity, and opposite intensity). In the ‘same intensity’ condition the listener’s audio–visual stream was replaced with a recording of the same listener producing a laugh response with the same intensity, but taken from a different point in the conversation. In the ‘opposite intensity’ condition the listener’s laugh response was replaced with a laugh response by the same listener but with the opposite intensity; that is, low and high intensity laugh responses were replaced with high and low intensity laugh responses, respectively. In the control condition participants viewed the original story-teller/listener interaction.

The dependent variable is the level of confidence that the interaction is genuine, i.e., that the interaction actually took place. The exact question is “Can you provide a rating of your confidence level between 0 (no confidence at all) and 10 (highly confident) that this is a genuine interaction?

**FIGURE 2:**
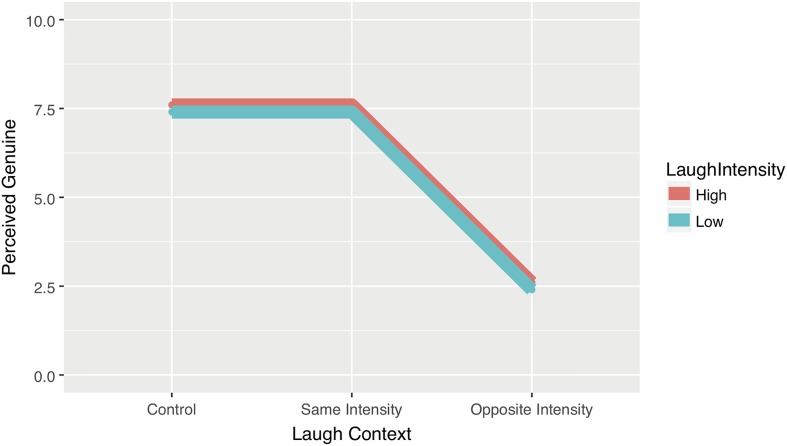
Hypothesised pattern of results for Experiment 1: Interchanged laughter leads to perceptions of genuine interactions when intensity is the same but not when intensity is different.

We adopt the statistical recommendations of Cumming (2012, 2014) and Cumming and Calin-Jageman (2017), using point estimates with confidence intervals and effect sizes to convey precision and the magnitude of the experimental effects. This approach does not change the fundamental frequentist philosophy in the statistics but alters the emphasis toward presenting effect sizes and away from point estimates of *p*-values through the use of confidence intervals. In addition, we have created estimated values for our hypotheses, these are arbitrary estimates in absolute terms but the pattern of results is based on the reasoning we have outlined. Our scale for the assessment of genuine interactions runs from 0 to 10. Aware of the central tendency and range restriction errors outlined by Saal et al. (1980) we assume that even when participants strongly believe that an interaction is genuine they will be reluctant to suggest a rating of 10; we therefore place our estimate of belief in a genuine interaction at the upper quartile, 7.5, and our estimate of a not genuine interaction at the lower quartile of 2.5. For the two control conditions, which are genuine interactions, we estimate they will both be viewed as genuine: thus, we predict that participants will give a maximum genuineness score of 7.5 for original recordings regardless of intensity of the laughable context/laugh response. Similarly, we predict that maximum genuineness scores will be given in the interchanged conditions in which a laugh is replaced with a laugh of the same intensity. However, where the interchange involves swapping laughs of different intensity (i.e., the replacement laugh does not match the laughable context), we predict that such interactions will be seen as not genuine and will be assigned the lowest genuineness score of 2.5. **Figure 2** displays these estimates in a graphical form.

## Experiment 1

### Method

#### Participants

One hundred and one participants (40 women, 61 men, mean age = 33.16 years, age range = 20–68 years) were recruited using Amazon’s Mechanical Turk, a crowdsourcing website which produces high quality data that are at least as reliable as those obtained through traditional methods (Casler et al., 2013; Paolacci and Chandler, 2014).

#### Materials

The general stimuli generation has already been outlined. In this experiment, we generated stimuli from two of the four high intensity laughable contexts and two of the four low intensity laughable contexts. These laughable contexts were paired with their original laugh responses to create two stimuli for the high intensity control condition and two stimuli for the low intensity control condition. Two different high intensity laugh responses were randomly selected and paired with the two high intensity laughable contexts to create two stimuli for the interchanged same-high-intensity condition. Two different low intensity laugh responses were randomly selected and paired with the two low intensity laughable contexts to create two stimuli for the interchanged same-low-intensity condition. Two different high intensity laugh responses were randomly selected and paired with the two low intensity laughable contexts to create two stimuli for the interchanged opposite-high-intensity condition. Finally, two different low intensity laugh responses were randomly selected and paired with the two high intensity laughable contexts to create two stimuli for the interchanged opposite-low-intensity condition. This gave a total of 12 stimulus clips in 6 conditions, 2 in each condition.

### Procedure

All participants viewed all 12 clips, and provided ratings of level of confidence that the interaction was genuine for each stimulus.

### Results

**FIGURE 3:**
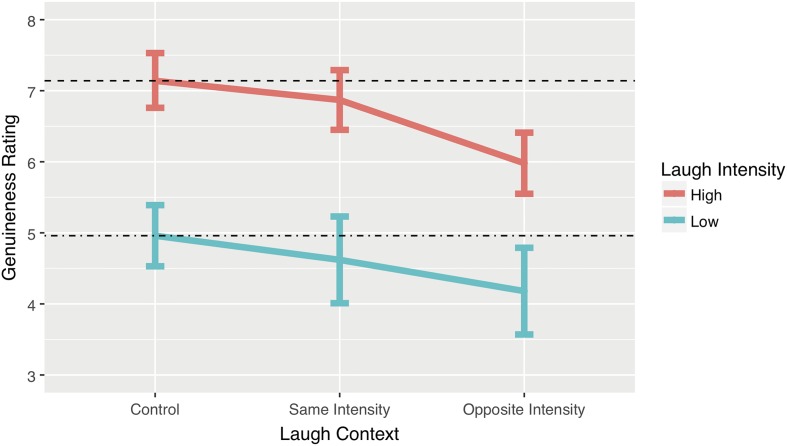
Point estimates derived from the model for each of the conditions. Red and blue lines plot genuineness ratings of interactions containing high and low intensity laughter, respectively. Although interactions containing low intensity laughter were consistently judged less genuine than those containing high intensity laughter, both sets of data reveal a similar trend. In both cases there was no significant change in genuineness ratings when laughter in an original interaction was replaced with similar intensity laughter. However, replacements with laughter of the opposite intensity result in a measurable reduction in genuineness ratings. Error bars represent 95% Confidence Intervals.

The general pattern of the results (**Figure 3**) show that participants’ genuineness ratings were unaffected when the listener’s laugh was replaced with a same-intensity laugh from a different point in the conversation. However, replacing a laugh with an opposite-intensity laugh resulted in a measurable reduction in genuineness ratings. An unexpected finding was that real interactions containing low intensity laughter (**Figure 3**, lower line) were consistently judged as less genuine than real interactions containing high intensity laughter (**Figure 3**, upper line). We address the implications of this finding in the discussion.

The analyses were performed using multi-level models to generate point estimates of the mean with confidence intervals. The multi-level approach accounts for the dependency in the data due to using the same participants to rate more than one video clip, and avoids underestimation of the standard errors (Quené and van den Bergh, 2004; McKeown and Sneddon, 2014). Point estimates are labelled *M*_est_, and arithmetic means and standard deviations are provided in **Table 1**. The R (R Core Team, 2017) package lme4 (Bates et al., 2015) was used for the multilevel models and generation of profile confidence intervals.

**Table 1:** Table of the fixed effects factors for the multi-level model of laugh responses for Experiment 1.

	Condition	*b*	*SE*	*t*	95% CI	Point estimate	*M*	*SD*
Intercept	Control High intensity	7.14	0.20	36.10	6.76, 7.53	7.14	7.14	2.17
High context	High context Same intensity	-0.28	0.22	-1.26	-0.70, 0.15	6.87	6.87	2.22
Low context	Low context Opposite intensity	-1.16	0.22	-5.29	-1.59, -0.73	5.98	5.98	2.36
Low intensity	Control Low intensity	-2.18	0.22	-9.93	-2.61, -1.75	4.96	4.96	2.68
LowC^∗^LowI	Low context Same intensity	0.83	0.31	2.66	0.22, 1.44	4.62	4.62	2.71
HighC^∗^LowI	High context Opposite intensity	-0.5	0.31	-1.61	-1.11, 0.11	4.18	4.18	2.3


Genuineness scores in the high intensity control condition were very similar to our hypothesised estimates (*M*_est_ = 7.14). The lowest level of reported genuineness (*M*_est_ = 4.18, for high intensity laughter inserted into a low intensity context) was considerably more than the hypothesised 2.5, the lowest estimate of genuineness predicted. The first important difference with our hypothesised estimates is that the low intensity control condition was judged as less genuine (*M*_est_ = 4.96, 95% CI [4.53, 5.39]) than the high intensity control condition (*M*_est_ = 7.14, 95% CI [6.76, 7.53]), even though both conditions involved genuine interactions. This suggests that something in the nature of the low intensity laugh responses and laughable contexts results in the overall interaction being judged as less genuine than interactions that contain high intensity laugh responses and laughable contexts. As a result, we will treat the low intensity laugh results and high intensity laugh results independently. We, therefore, use the ratings for the control interactions as our reference point estimate in the models against which the experimental conditions can be compared. Participant variance was modelled as a random parameter using a random intercept multilevel model (participant variance = 1.51, *SD* = 1.23; residual variance = 4.89, *SD* = 2.21). Fixed effect statistics are provided in **Table 1**.

#### High Intensity Laughter

##### Same context

When laughter from high intensity laughable contexts were swapped for laughter taken from other high intensity laughable contexts to produce stimuli of interactions that never occurred, we found that genuineness ratings were similar to those in the control condition (*M*_est_ = 6.87, 95% CI [6.45, 7.29]). The model *b* coefficient provides the best effect size in the units of the study between the high intensity control condition and the same intensity condition, representing a reduction of perceived genuineness of the interaction by 0.28 on the 0–10 scale. Given the difficulty of choosing a standardised effect size measure for local effects within mixed-effects regression models (Selya et al., 2012), we adopt a technique used by Friedmann et al. (2008) where mean difference scores are calculated from the model generated point estimates and the control group standard deviation is used to provide a measure of Cohen’s *d*; here *d* = 0.13. The Common Language Effect Size (CLES) (McGraw and Wong, 1992; Lakens, 2013) indicates that, after controlling for individual differences, the likelihood that a person rates the control interaction stimuli as more genuine than the swapped laugh stimuli is 54% (50% corresponds to no difference). Thus, interchanging high intensity laughs has little or no effect on ratings of the genuineness of an interaction.

##### Different context

In contrast to the same-context condition, when high intensity laugh responses were inserted into low intensity laughable contexts, the mean genuineness ratings were considerably lower (M_est_ = 5.98, 95% CI [5.55, 6.41]). A *b* coefficient of -1.16 provides a study unit effect size estimate of the difference between the high-intensity control condition and the opposite-intensity condition–in this case a low-intensity laughable context with a high-intensity laugh. It represents a reduction of perceived genuineness of the interaction by 1.16 on the 0–10 scale. Cohen’s *d* (0.54) and the CLES indicate that, after controlling for individual differences, the likelihood that a person rates the swapped laugh stimuli as less genuine than the control interaction stimuli is 65%. In terms of Cohen’s *d* rule of thumb this would be a medium effect size (Cohen, 1988).

#### Low Intensity Laughter

##### Same context

When laughter in low intensity laughable contexts is replaced with laughter taken from other low intensity laughable contexts we find that genuineness ratings are once again similar to those in the control condition (M_est_ = 4.62, 95% CI [4.01, 5.23]). A *b* coefficient of 0.83 corresponds to a reduction of perceived genuineness of the interaction by 0.34 on the 0–10 scale. Cohen’s *d* (0.13) and the CLES indicate that, after controlling for individual differences, the likelihood that a person rates the control interaction stimuli as more genuine than the swapped laugh stimuli is 54%. Thus, interchanging a low intensity laugh has little or no effect on ratings of the genuineness of an interaction.

##### Different context

When low intensity laugh responses were inserted into high intensity laughable contexts the mean genuineness ratings were the lowest observed in this experiment (M_est_ = 4.18, 95% CI [3.57, 4.79]). A *b* coefficient of -0.5 corresponds to a reduction of the perceived genuineness of the interaction by 0.78 on the 0–10 scale. Cohen’s *d* (0.29) and the CLES indicate that the likelihood a person rates the control interaction stimuli as more genuine than the swapped laugh stimuli is 58%. In terms of Cohen’s *d* rule of thumb this would be a small effect.

Acknowledging the historical context of the discipline and the role of null hypothesis significance testing within this context, and due to the importance of the issues raised by Gelman and Stern (2006) we also present the results of this analysis using a 2 × 3 ANOVA. We present the main effects and interaction effect of the ANOVA but encourage researchers to give more prominent attention toward the simple main effects using the multi-level model generated point estimates and confidence interval approach for detailed analysis with respect to theoretical concerns. There is a significant main effect of laughable context *F*(2,1206) = 9.38, *p* < 0.001, η^2^ = 0.01. There is also a significant main effect of laugh intensity *F*(1,1206) = 204.04, *p* < 0.001, η^2^ = 0.14. Finally, there is a significant interaction of laughable context and laugh intensity *F*(2,1206) = 7.1, *p* < 0.001, η^2^ = 0.01.

### Discussion

The results of Experiment 1 provide support for our hypothesis that laughter of the same intensity can be interchanged with other laughs of similar intensity without affecting the apparent genuineness of the interaction. Where the level of laughter intensity does not match the context into which it is inserted, effects are observed – a medium effect size in the case of high intensity laugh response and a small effect size in the case of low intensity laugh responses. The finding that laughs of similar intensity are wholly interchangeable without affecting an interaction’s perceived genuineness provides a proof of concept for our hypothesis that laughter is inherently ambiguous; as such this finding poses a challenge for the representational model of laughter and is consistent with the affect-induction model.

An additional important finding is the overall reduction in genuineness associated with low intensity laughs. The genuine low-intensity situation was judged to be less genuine than the worst case condition that contained a high intensity laugh response. It appears that even when strong laughter occurs with no expectation cues, these interactions are deemed to be more genuine than real interactions that contain low intensity laughter.

There are some limitations to this study. We only used four of the eight actual laugh contexts selected, and we cannot rule out the possibility that the present findings were due to specific features of the contexts displayed in the stimuli. Another caveat is that all the stimuli were judged by each participant in this experiment, allowing for the possibility that judgements of a laugh were made relative to responses to other laughs paired with the same context. These limitations are addressed in Experiment 2.

## Experiment 2

Experiment 2 was a direct replication of Experiment 1, but with additional manipulations that address the above limitations. The number of stimuli used was increased to the maximum possible given the selection of eight usable contexts. In addition, we also wished to remove the possibility that responses to previous stimuli combinations were interfering with judgements being made about the genuineness of a given interaction. We excluded this possibility by ensuring that each participant saw each laugh context only once. We hypothesised that the results would follow the same pattern observed in Experiment 1.

### Method

#### Participants

As we were not getting more than one rating per context from the participants in this experiment we increased the sample size and recruited 404 participants (153 women, 251 men, mean age = 33.16 years, age range = 20–68 years) via Amazon’s Mechanical Turk.

#### Stimulus Generation

The stimuli were created using the same eight laughs selected for use in Experiment 1. The main difference was that on this occasion we created all possible stimulus combinations with the eight laugh contexts. This gave a total of 64 stimulus clips. The same 2 (laugh response intensity) × 3 (laughable context) design was used in the presentation of the stimuli, giving six conditions. There were four original high intensity laughter clips in the high intensity control condition; 4 original low intensity clips in the low intensity control condition; 12 high intensity clips in the high-same-intensity condition; 12 low intensity clips in the low-same-intensity condition; 16 low intensity laughter clips in the high-opposite-intensity condition; 16 high intensity clips in the low-opposite-intensity condition.

### Procedure

The procedure was the same as in Experiment 1 except on this occasion we showed eight videos to each participant and ensured that no laughter-inducing context was repeated in a given experimental run. We randomly selected one of the laughter stimuli for each laugh context for presentation to the participants. The condition cells are necessarily imbalanced due to the nature of the stimulus generation; there are only eight genuine interactions to create the control conditions, only 24 stimuli can be used in the same intensity conditions as the control conditions cannot be used, and there are 32 possible combinations for the opposite intensity conditions. These differences in number of stimuli and number of participants across the cell sizes are largely accommodated by the use of multi-level models, which are more robust to unequal cell sizes than repeated measure ANOVA models.

### Results

**FIGURE 4:**
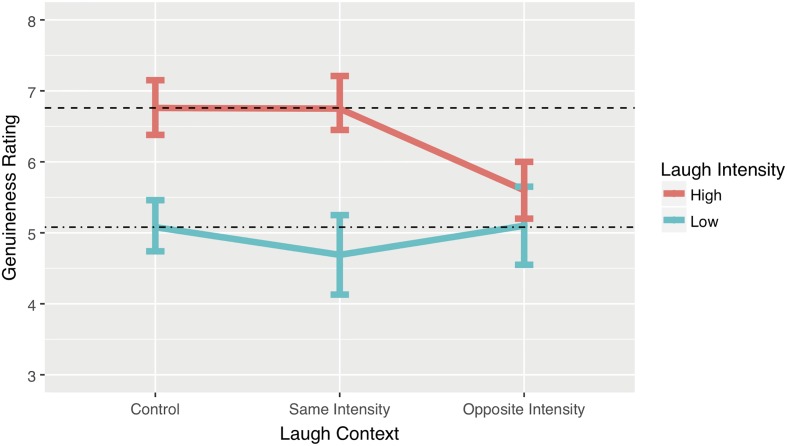
Point estimates derived from the model for each of the conditions. Error bars represent 95% Confidence Intervals.

The absolute numerical estimates for the conditions were slightly different from Experiment 1, but the pattern was the same (see **Figure 4**): in the control condition using low intensity laughter genuineness was again rated lower (*M*_est_ = 5.08, 95% CI [4.59, 5.57]) than in the control condition using high intensity laughter (*M*_est_ = 6.76, 95% CI [6.38, 7.15]). Once again, we used a multi-level model to generate point estimates of the mean with profile confidence intervals. Participant variance was modelled as a random parameter using a random intercept multilevel model (participant variance = 1.69, *SD* = 1.3; residual variance = 5.93, *SD* = 2.43). Fixed effect statistics are provided in **Table 2**.

**Table 2:** Table of the fixed effects factors for the multi-level model of laugh responses for Experiment 2.

	Condition	*b*	*SE*	*t*	95% CI	Point estimate	*M*	*SD*
Intercept	Control High intensity	6.76	0.20	34.67	6.38, 7.15	6.76	6.76	2.17
High context	High context Same intensity	-0.02	0.21	-0.13	-0.44, 0.38	6.75	6.7	2.39
Low context	Low context Opposite intensity	-1.16	0.20	-5.69	-1.56, -0.76	5.60	5.57	2.94
Low intensity	Control Low intensity	-1.69	0.25	-6.74	-2.18, -1.20	5.08	5.1	2.8
LowC^∗^LowI	Low context Same intensity	0.78	0.29	2.74	0.22, 1.34	4.69	4.72	2.81
HighC^∗^LowI	High context Opposite intensity	0.05	0.28	0.19	-0.50, 0.61	5.1	5.13	2.86


#### High Intensity Laughter

##### Same context

When laugh responses from high intensity laughable contexts are swapped for laugh responses taken from other high intensity laughable contexts we find that genuineness ratings are almost identical to those in the control condition (M_est_ = 6.75, 95% CI [6.45, 7.21]). A *b* coefficient of -0.02 represents a reduction of perceived genuineness of the interaction relative to the control condition by 0.02 on the 0–10 scale. Cohen’s *d* (0.01) and the CLES indicate that the likelihood that a person rates the control interaction stimuli as more genuine than the swapped laugh stimuli is 50%; this suggests that interchanging high intensity laughs has no effect on ratings of the genuineness of an interaction.

##### Different context

When high intensity laughter was inserted into low intensity laughable contexts the mean genuineness ratings were considerably lower (M_est_ = 5.60, 95% CI [5.2, 6]). A *b* coefficient of -1.16 represents a reduction of perceived genuineness of the interaction by 1.16 on the 0–10 scale. Cohen’s *d* (0.47) and the CLES indicate that the likelihood a person rates the swapped laugh stimuli as less genuine than the control interaction stimuli is 63%. In terms of Cohen’s *d* rule of thumb this would be a medium effect size.

#### Low Intensity Laughter

##### Same context

When laughter from low intensity laughable contexts is replaced with laughter from other low intensity laughable contexts we obtain a pattern of ratings (*M*_est_ = 4.69, 95% CI [4.13, 5.25]) similar to experiment 1. A *b* coefficient of 0.78 represents a difference in perceived genuineness by 0.78 between the two conditions. Cohen’s *d* (0.14) and the CLES indicate that the likelihood that a person rates the swapped laugh stimuli as less genuine than the control interaction stimuli is 54%. Thus, interchanging low intensity laughs has little or no effect on ratings of the genuineness of an interaction.

##### Different context

On this occasion when low intensity laughter was inserted into high intensity laughable contexts the mean genuineness ratings (M_est_ = 5.1, 95% CI [4.55, 5.65]) were at similar levels to the control reference condition. A *b* coefficient of 0.05 indicates an increase in the perceived genuineness of the interaction by 0.05 on the 0–10 scale. Cohen’s *d* (0.02) and the CLES indicate that the likelihood a person rates the swapped laugh as less genuine than the control interaction stimuli is 51%. Interchanging a low intensity laugh into a high intensity laughable context has no effect on ratings of the genuineness of an interaction. Interchanging a low intensity laugh into a high intensity laughable context has no effect on ratings of the genuineness of an interaction.

Once again, we present the main effects and interaction effect of the ANOVA but encourage researcher to give more prominent attention toward the simple main effects using the multi-level model generated point estimates and confidence interval approach for detailed analysis with respect to theoretical concerns. There is a significant main effect of laughable context *F*(2,3226) = 31.15, *p* < 0.001, η^2^ = 0.01. There is also a significant main effect of laugh intensity *F*(1,3226) = 138.75, *p* < 0.001, η^2^ = 0.04. Finally, there is a significant interaction of laughable context and laugh *F*(2,3226) = 7.01, *p* < 0.001, η^2^ = 0.004.

### Discussion

The pattern of results for high intensity laughter in Experiment 2 is even more similar to the original hypothesis than those observed in Experiment 1. Genuineness ratings are almost identical for the control and ‘same intensity’ conditions with a drop off in genuineness ratings for the ‘opposite intensity’ condition. This provides strong support for the original hypothesis in the case of high intensity laughter. Thus, interchanging high intensity laughter seems to have little or no effect on the perceived level of genuineness of the interaction.

The pattern of results for low intensity laughter is somewhat different to those from Experiment 1, the key difference being that genuineness ratings do not drop off in the ‘opposite intensity’ condition. This is the only condition that failed to replicate results of Experiment 1. It may be that low intensity laughter is inherently more ambiguous than high intensity laughter; McKeown et al. (in preparation) argue that low intensity laughter has many more functions than high intensity laughter, with the latter more closely related to the assessment of humour production. Although low intensity laughter is seen as being part of less genuine interactions, these interactions are not rated as ‘not genuine’; rather they occupy the midpoint on the scale. It may be that high intensity laughter may more unequivocally indicate genuineness whereas low intensity laughter can be interpreted in many different ways–thus increasing the likelihood it will be viewed as being consistent with the context it is inserted into.

Another way of understanding the results may be that the nature of these laughter effects in the two opposite scenario interactions leads to quite different interpretations of the social interaction. In one a high intensity laugh response occurs despite the story-teller not providing contextual cues that a high intensity laugh was expected. Laughter in such a scenario might reasonably be interpreted as an over effusive laugh response, and consequently the interaction deemed to be less genuine. In the other case the contextual cues did indicate that a high intensity laugh response was expected but was greeted with a low intensity laugh–an interactional situation that may be observed to be an insult or social rejection–these situations were rated as the least genuine of all.

## General Discussion

The experiments reported here address an important question regarding the function of laughter; namely, is it used to signal the laugher’s underlying emotional state or is its function to influence the listener’s emotional state? This question addresses a current debate on the function of human laughter. While the representational model of laughter proposes that laughter encodes information about the laugher’s emotion(s), which is then decoded by the listener, the affect-induction model depicts laughter as a communicative tool with which to influence the emotions of the listener. The representational model views laughter as an unambiguous signal of the laugher’s emotional state; the affect-induction model, on the other hand, proposes that laughter interpretation is determined by situational factors. Thus, unlike the representational model, the affect-induction model would predict that laughter is an ambiguous signal subject to contextual influences. Our experiments sought to pit the two models against each other by switching laughter in an original interaction with different laughter from a different part of the interaction. The representational model would predict that participants should be able to differentiate between original interactions and interactions in which laughter has been switched; the affect-induction model, on the other hand, would predict that participants would not be able to make this distinction. Furthermore, we argued that any inability to differentiate between original interactions and those in which laughter has been switched should be restricted to original and replacement laughs of similar intensity.

The results of Experiment 1 show that participants’ ratings of an interaction’s genuineness were unaffected when the listener’s laughter was swapped for another instance of laughter of similar intensity and from the same listener, but from a different point of the interaction. However, replacing listener laughter with laughter of a different intensity resulted in participants rating the story telling interaction as less genuine. This demonstration of the ambiguous nature of same intensity laughter is consistent with the affect-induction model, which would argue that laughter is necessarily ambiguous as regards the laugher’s underlying emotional state.

Experiment 1 had a number of limitations. For example, not all possible context – laughter combinations were used to generate the stimuli. Furthermore, participants would have viewed each context several times, with each containing a different laugh stimulus; thus it is feasible that responses to a given context would have been tempered by previous responses to the same context (but paired with different laughter). Experiment 2 overcame these limitations by using the full range of context-laughter combinations and ensuring that participants were presented with each context only once. The results of Experiment 2 replicated those of Experiment 1 in all but one condition. When laughter (high or low intensity) was switched for laughter of a similar intensity, participants’ genuineness judgements were unaffected. When high intensity laughter was inserted into a low intensity context, participants judged the interaction as less genuine. However, this was not the case when low intensity laughter was inserted into a high intensity context; rather, the interaction was judged to be as genuine as the control and same-intensity conditions. It has been proposed that low intensity laughter is functionally more complex and inherently more ambiguous than high intensity laughter (McKeown et al., in preparation), and this may explain why inserting a low intensity laugh into a high intensity context did not result in the interaction appearing less genuine.

An interesting finding was that interactions retaining their original low intensity laughter were consistently judged less genuine than those retaining their original high intensity laughter. Previous research highlighting physiological differences between spontaneous and volitional laughter production (Bryant and Aktipis, 2014) might offer an explanation for this finding. It would be reasonable to assume that differences in the sounds of spontaneous and volitional laughter are likely to be magnified with increasing laughter intensity, and that it should be more difficult to differentiate between low intensity spontaneous and volitional laughter. The low genuineness ratings of the low intensity laughs might, therefore, be a consequence of the relative difficulty in correctly identifying spontaneous low intensity laughter. In other words, if participants are uncertain about a laugh’s spontaneity they will be more likely to identify the interaction as being less genuine. This may explain why the observed reduction in genuineness scores in Experiment 1 when low intensity laughter was combined with a different intensity context did not generalise to Experiment 2. Recall that Experiment 2 was motivated by a desire to use more laughter stimuli than in Experiment 1. A consequence of this was that a higher proportion (75%) of low intensity laughs in Experiment 2 were voiced compared to Experiment 1 (50%). If voiced laughter is judged as more genuine, then the higher proportion of voiced laughter in Experiment 2 might explain the different results in this condition across experiments.

The results of our experiments provide compelling evidence that laughter is an inherently ambiguous stimulus, and that its interpretation is largely determined by the context in which it occurs. As such these results support the affect-induction model. While it is the case that we often talk of laughter ‘types,’ such as sardonic laughter, joyful laughter, taunting laughter, and schadenfreude laughter, our results suggest that different instances of same-intensity laughter are largely interchangeable and that their specific meaning may be largely determined by the context in which they occur. This flexibility is reminiscent of recent, similar findings relating to the facial expression of emotions. Historically, it has been widely accepted that the facial expression of emotion is underpinned by emotion-specific facial musculature activation patterns (Ekman and Friesen, 1971); from this perspective a sad facial expression and a fearful facial expression are associated with distinct combinations of facial movements. However, recent developments suggest that, despite all their information value, facial expressions can be ambiguous and that their meaning is largely dependent on contextual information beyond the face (Aviezer et al., 2011; Barrett et al., 2011; Hassin et al., 2013). Our results suggest that the important role of context in the perception of facial expressions also applies to the interpretation of laughter.

## Conclusion

We tested which of two models, representational or affect-induction, best describes the function of laughter. We devised novel experiments such that the two models made opposite predictions, and found that the results are consistent with the affect-induction model’s prediction.

## Ethics Statement

The experiments were carried out in accordance with the recommendations of the School of Psychology Ethics Committee, Queen’s University Belfast, with written informed consent from all subjects. All subjects gave written informed consent in accordance with the Declaration of Helsinki. The protocol was approved by the School of Psychology Ethics Committee, Queen’s University Belfast.

## Author Contributions

All authors listed have made a substantial, direct and intellectual contribution to the work, and approved it for publication.

## Conflict of Interest Statement

The authors declare that the research was conducted in the absence of any commercial or financial relationships that could be construed as a potential conflict of interest.
